# Airborne transmission risks of tuberculosis and COVID-19 in schools in South Africa, Switzerland, and Tanzania: Modeling of environmental data

**DOI:** 10.1371/journal.pgph.0002800

**Published:** 2024-01-18

**Authors:** Nicolas Banholzer, Remo Schmutz, Keren Middelkoop, Jerry Hella, Matthias Egger, Robin Wood, Lukas Fenner

**Affiliations:** 1 Institute of Social and Preventive Medicine, University of Bern, Bern, Switzerland; 2 Institute of Infectious Disease and Molecular Medicine, University of Cape Town, Cape Town, South Africa; 3 Desmond Tutu Health Centre, Department of Medicine, University of Cape Town, Cape Town, South Africa; 4 Ifakara Health Institute, Dar-es-Salaam, Tanzania; 5 Centre for Infectious Disease Epidemiology & Research, School of Public Health & Family Medicine, University of Cape Town, Cape Town, South Africa; 6 Population Health Sciences, Bristol Medical School, University of Bristol, Bristol, United Kingdom; Epicentre MSF, SWITZERLAND

## Abstract

The COVID-19 pandemic renewed interest in airborne transmission of respiratory infections, particularly in congregate indoor settings, such as schools. We modeled transmission risks of tuberculosis (caused by *Mycobacterium tuberculosis*, *Mtb*) and COVID-19 (caused by SARS-CoV-2) in South African, Swiss and Tanzanian secondary schools. We estimated the risks of infection with the Wells-Riley equation, expressed as the median with 2.5% and 97.5% quantiles (credible interval [CrI]), based on the ventilation rate and the duration of exposure to infectious doses (so-called quanta). We computed the air change rate (ventilation) using carbon dioxide (CO_2_) as a tracer gas and modeled the quanta generation rate based on reported estimates from the literature. The share of infectious students in the classroom is determined by country-specific estimates of pulmonary TB. For SARS-CoV-2, the number of infectious students was estimated based on excess mortality to mitigate the bias from country-specific reporting and testing. Average CO_2_ concentration (parts per million [ppm]) was 1,610 ppm in South Africa, 1,757 ppm in Switzerland, and 648 ppm in Tanzania. The annual risk of infection for *Mtb* was 22.1% (interquartile range [IQR] 2.7%-89.5%) in South Africa, 0.7% (IQR 0.1%-6.4%) in Switzerland, and 0.5% (IQR 0.0%-3.9%) in Tanzania. For SARS-CoV-2, the monthly risk of infection was 6.8% (IQR 0.8%-43.8%) in South Africa, 1.2% (IQR 0.1%-8.8%) in Switzerland, and 0.9% (IQR 0.1%-6.6%) in Tanzania. The differences in transmission risks primarily reflect a higher incidence of SARS-CoV-2 and particularly prevalence of TB in South Africa, but also higher air change rates due to better natural ventilation of the classrooms in Tanzania. Global comparisons of the modeled risk of infectious disease transmission in classrooms can provide high-level information for policy-making regarding appropriate infection control strategies.

## Introduction

Increasing evidence underscores the role of airborne transmission in respiratory infections like SARS-CoV-2 [1–5], which has been affirmed by institutions such as the Centers for Disease Control and Prevention and the World Health Organization [6, 7]. Airborne transmission can occur when an infectious person exhales infectious respiratory particles into the air, which enter a susceptible person’s respiratory tract after inhalation of these particles from contaminated air. Pathogens are predominantly found in smaller particles <5 *μ*m [8], which can remain in the air for several hours, travel further, and reach deeper into the respiratory tract [2]. Current understandings of airborne transmission of respiratory infections emanate from the groundwork in tuberculosis (TB) research [9]. Caused by the bacterium *Mycobacterium tuberculosis* (*Mtb*), TB is a strictly airborne infection [8] and was the leading cause of death globally for any infectious disease until the COVID-19 pandemic.

Airborne transmission of respiratory infections (including *Mtb* and SARS-CoV-2) is more likely in indoor settings such as healthcare facilities, restaurants, offices, and schools [10]. School closures were most intensely debated during the COVID-19 pandemic because it was unclear how much they affected community and household transmission [11–13]. Infection control measures could mitigate transmission risks [12, 14, 15] and allow schools to remain open. In particular, previous studies have shown that the risk of indoor transmission decreases with mechanical ventilation (i.e., a mechanical ventilation system that circulates fresh air) and natural ventilation (i.e., by opening windows and doors) [16–19], which replace contaminated indoor air with fresh outdoor air. The ventilation (or replacement) rate can be monitored using indoor carbon dioxide (CO_2_) as a tracer gas [20]. Ventilation rate is an important parameter in modeling the risk of airborne infection [15, 21–23].

This study estimated and compared the transmission risk of SARS-CoV-2 and *Mtb* between three secondary schools in South Africa, Switzerland, and Tanzania. We used indoor CO_2_ measurements from different schools in each country over prolonged periods. We estimated the risk of infection during school hours using the Wells-Riley equation [24], as modified by Rudnick and Milton [25], by making assumptions about the generated rate of infectious doses and the proportion of contagious students in the classroom.

## Methods

### Environmental, infrastructure and room occupancy

An overview of the data used in this study is presented in Table 1. Our study is primarily based on data collected in secondary schools in Cape Town (South Africa, age 15–19 years) [22], the canton of Solothurn (Switzerland, age 15–19 years) [26], and Dar-es-Salaam (Tanzania, age 15–19 years) [21]. None of the schools used mechanical ventilation to ventilate classrooms. Instead, classrooms in South Africa and Switzerland were ventilated through opening windows and in Tanzania through constant outdoor air exchange (no windows). Room occupancy varied between schools, with 30 students in South Africa, 20 in Switzerland, and 50 in Tanzania. We filtered the CO_2_ data collected in each school according to the times students occupied the rooms (excluding lessons outside rooms, breaks, and school-free hours).

**Table 1:** Overview of study setting and collected data.

	South Africa	Switzerland	Tanzania
Location	Cape Town	Canton of Solothurn	Dar-es-Salaam
Year	2013	2023	2015
Volume of classrooms (m^3^), *vol*	180	233	162
Number of students (average), *n*	30	20	50
Age of students (range)	15–19	13–15	15–19
CO_2_ (ppm), *C*			
Mean, overall	1,610	1,757	648
Mean (SD), daily mean	1,421 (451)	1,702 (370)	600 (58)
Mean (SD), daily max	2,728 (1,131)	2,373 (539)	951 (587)
Ventilation rate (L s^-1^), *Q*			
Mean (SD), daily max *C*	2.47 (1.81)	2.29 (0.88)	12.36 (7.46)
Air change rate (h^-1^), *ACH*			
Mean (SD), daily max *C*	1.48 (1.08)	0.71 (0.27)	13.74 (8.29)
Rebreathed air fraction (%), $\bar{f}$			
Mean, overall *C*	3.8	4.3	0.8
Mean (SD), daily mean *C*	3.2 (1.4)	4.1 (1.2)	0.6 (0.2)
Sampling duration and frequency	91 days during a school year	35 days over 7 weeks (Jan–Mar)	5 days over 2 weeks (Jul)
Sampling setting	Several schools and classrooms	One school and several classrooms	Four schools and classrooms
Reference	Richardson *et al*. [22]	Banholzer *et al*. [26]	Hella *et al*. [21]

CO_2_, carbon dioxide; IQR, interquartile range; ppm, parts per minute; SD, standard deviation

### Statistical analyses and modeling

We used the Wells-Riley equation as modified by Rudnick and Milton [25] to model the risk of airborne transmission for *Mtb* and SARS-CoV-2. The risk of infection *P* (in %) is estimated as

$$P=\frac{D}{S}=1-\exp\left(-\frac{\bar{f}Iqt}{n}\right),$$

where *D* is the number of diseased cases, *S* is the number of susceptibles, $\bar{f}$ is the time-weighted average fraction of rebreathed air (in %), *I* is the number of infectious students; *n* is the total number of students in the room, *q* is the rate of infectious doses (quanta) generated by infectious individuals (in quanta h^-1^), and *t* is the duration of exposure (in h). We computed the average rebreathed air fraction as $\bar{f}=(\bar{C}-C^{O})/C^{a}$ using the average of the indoor CO_2_ measurements (*C* in parts per million [ppm]) and assuming a CO_2_ level of *C*^***o***^ = 400 ppm in the outdoor air [20, 22, 27–30] as well as *C*^*a*^ = 31,500 ppm in the exhaled air [25, 31].

In addition, we computed the ventilation rate (in L s^-1^ per individual) as $Q=G/(\overset{ˇ}{C}-C^{o})$ and the air change rate (in air changes h^-1^) as ACH=(3,600⋅Q⋅n)/V using the the daily maximum of the CO_2_ measurements $\overset{ˇ}{C}$ as the steady-state levels [20]. We assumed a CO_2_ generation rate of $G=1/(60\cdot 10^{6})\cdot b\cdot C^{a}=0.0042$ L/s per individual in a lecture classroom [31] as well as a breathing rate of *b* = 8 L/min per individual [25]. Of note, the time points and classrooms of the CO_2_ levels collected in South Africa and Tanzania were usually unknown. Therefore, the daily maximum CO_2_ levels $\overset{ˇ}{C}$ were determined with a quantile-based approach by evaluating the inverse of the empirical distribution function *F*^−1^(*y*) in South Africa ${\overset{ˇ}{c}}_{SA}=F_{SA}^{-1}(y_{CH})$ and Tanzania ${\overset{ˇ}{c}}_{TZ}=F_{TZ}^{-1}(y_{CH})$ at the quantiles of the daily maxima in Switzerland ${y_{CH}=F}_{CH}({\overset{ˇ}{c}}_{CH})$.

We estimated the annual risk of transmission for *Mtb* and the monthly risk for SARS-CoV-2. We assumed t^year^ = 919 and t^month^ = 919/12 = 77 school-hours, corresponding to the OECD average for lower secondary schools [32]. We used Monte Carlo simulation to estimate uncertainty in the quanta generation rate *q* and the number of infectious persons per classroom *I* (see below). We performed 4,000 simulations and estimated the risk of infection *P* based on randomly drawn samples from the distributions of *q* and *I*. We summarized our simulation results with boxplots showing the median, interquartile range (IQR or 50%-credible interval [CrI]), and 95%-CrI. We performed all analyses with R software (version 4.2.2).

### Modeling assumptions

#### Infectious quanta

The range of estimates for the quanta generation rate *q* is large for both SARS-CoV-2 and *Mtb* [33–38]. There are two approaches to estimating *q*. One approach is to solve the Wells-Riley equation for *q* using data on the number of susceptible and diseased cases [35–38]. Another more recently developed approach is to predict *q* from the viral or bacterial load in sputum [34] using the equation

$$q=c_{v}\cdot c_{i}\cdot IR\cdot V_{d},$$

where *c*_*v*_ is the viral or bacillay load (RNA copies mL^-1^ or CFU mL^-1^), *c*_*i*_ is a conversion factor (quanta RNA copies^-1^ or quanta CFU^-1^), *IR* is the inhalation rate (m^3^ h^-1^), and *V*_*d*_ is the droplet volume concentration expelled by the infectious person (mL m^3^). The product of *IR*×*V*_*d*_ is the droplet emission rate.

We applied the predictive approach using the viral load distributions and conversion factors for SARS-CoV-2 and *Mtb* as reported by Mikszewski et al. for the original SARS-CoV-2 strain and untreated TB patients [33]. We assumed the *IR* of a sitting person [39] and used the droplet emission rate of a loud-speaking person [40] to estimate the droplet emission rates for other activity levels by assuming the relative *V*_*d*_ for breathing, speaking, and loud speaking [41]. We considered three scenarios for the time-weighted quanta generation rates in the classroom: low (70% breathing, 25% speaking, 5% loud speaking), medium (50% breathing, 40% speaking, 10% loud speaking), and high activity (30% breathing, 50% speaking, 20% loud speaking). The quanta generation rates corresponding to these scenarios are shown in Table 2. They are roughly consistent with estimates from the literature [33–38]. For example, our medium scenario for *Mtb* assumes a *q* = 1.6 quanta h^-1^ (interquartile range 0.2 to 13.9), which covers the lower *q* = 0.27 quanta h^-1^ and upper *q* = 5.69 quanta h^-1^ reported by Andrews et al. [35] as well as other estimates from studies using the Wells-Riley approach to estimate *q* (S1 Table).

**Table 2:** Modeling assumption for the quanta generation rate. The quanta generation rates *q* are derived using the approach of Buonanno et al., which predicts *q* from sputum viral load. Viral loads and conversion factors for SARS-CoV-2 and *Mtb* are taken from Table 1 in Mikszewski et al. [33]. The droplet emission rates were recalculated based on values reported by Mikszewski *et al*. using the inhalation rate of a sitting person [39] for all activity levels. The activity-specific quanta generation rates were weighted by the proportion of time each activity occurs in the classroom. Three scenarios were considered: low (70% breathing, 25% speaking, 5% loud speaking), medium (50% breathing, 40% speaking, 10% loud speaking), and high activity (30% breathing, 50% speaking, 20% loud speaking).

	SARS-CoV-2	*Mtb*
Viral/bacillary load, log_10_ c_V_ mean (SD)	5.6 (1.2) RNA copies mL^-1^	5.5 (1.3) CFU mL^-1^
Conversion factor, c_i_	1.4 × 10^−3^ quanta RNA copies^-1^	2.0×10^−3^ quanta CFU^-1^
Droplet emission rate (mL h^-1^), *IR*×*V*_*d*_,		
Breathing	3.18×10^−2^	*same as for SARS-CoV-2*
Speaking	1.04×10^−3^	
Loud speaking	4.39×10^−3^	
Quanta generation rate (quanta h^-1^), *q* median (IQR) by activity level		
Breathing	0.6 (0.1–3.7)	0.7 (0.1–4.9)
Speaking	2.4 (0.4–15.7)	2.8 (0.4–20.9)
Loud speaking	17.8 (2.8–114.8)	20.2 (2.7–151.9)
Quanta generation rate (quanta h^-1^), *q* median (IQR) for different scenarios		
Low activity	1.0 (0.1–6.8)	1.1 (0.1–9.1)
Medium activity	1.4 (0.2–10.5)	1.6 (0.2–13.9)
High activity	2.3 (0.3–18.0)	2.6 (0.3–23.3)

*Mtb*, *Mycobacterium tuberculosis*; SD, standard deviation; *IR*, inhalation rate (m^3^ h^-1^); *V*_*d*_ droplet volume concentration (mL m^-3^); IQR, interquartile range

#### Infectious cases

For *Mtb*, we calculated the number of infectious students in each classroom from country-specific estimates of the prevalence of bacteriologically confirmed pulmonary TB in the 15–24 age group (Table 3). Estimates for Tanzania and South Africa were based on two national prevalence surveys conducted between 2011 and 2012 and between 2017 and 2019, respectively [42, 43]. We modeled the prevalence in these countries with a Normal distribution using the reported mean estimates and confidence intervals. Because a national prevalence survey was not available for Switzerland, we approximated the prevalence using the number of new TB notifications in the same age group [44], which implicitly assumes a duration of infectiousness of one year. We again modeled the prevalence with a Normal distribution using the mean and standard deviation of the reported notifications between 2015 and 2019.

**Table 3:** Modeling assumptions for infectious diseases incidence. Estimated prevalence (cases per 100,000 people) of *Mtb* in the young (age group of the 15 to 24-year-olds) and the general population [42–44]. Estimated incidence (new cases per 100,000 people) of SARS-CoV-2 using a consistent approach (see Methods) based on estimates of excess mortality [47] and time-updated, country-specifc infection-fatality ratios [48]. Reported incidence (new cases per 100,000 people) of SARS-CoV-2 in the young age group of the 10 to 20-year-olds) of Switzerland [51] and in the general population of South Africa [50] and Switzerland [51]. Incidence of SARS-CoV-2 in Tanzania is not shown because it has barely been reported [45, 46].

Infectious disease (Median [95%-CrI])	South Africa	Switzerland	Tanzania
***Mtb***			
Estimated prevalence			
Age group 15-24y	432 (232–632)	12 (5–20)	42 (11–73)
General population	852 (679–1,026)	57 (44–70)	293 (228–358)
Infectious students, *I*			
*Age group 15-24y*	0.130 (0.071–0.189)	0.002 (0.001–0.004)	0.021 (0.006–0.037)
General population	0.256 (0.203–0.308)	0.011 (0.009–0.014)	0.147 (0.114–0.179)
**SARS-CoV-2**			
Estimated Incidence			
IFR-based approach	1,557 (474–9,156)	360 (105–4,570)	928 (362–11,731)
Reported incidence			
Age group 10-20y		31 (2–754)	
General population	24 (0–190)	84 (4–1,713)	
Infectious students, *I*			
IFR-based approach	0.474 (0.143–2.730)	0.054 (0.013–0.111)	0.486 (0.297–1.157)
Age group 10-20y		0.006 (0.000–0.151)	
General population	0.007 (0.000–0.057)	0.017 (0.001–0.343)	

*Mtb*, *Mycobacterium tuberculosis*; CrI, credible

For SARS-CoV-2, we approximated the prevalence using the weekly incidence of COVID-19. However, the reporting and testing practices varied substantially across countries. For example, cases and deaths from COVID-19 were barely reported in Tanzania [45, 46]. Therefore, we employed a consistent approach for estimating the weekly incidence of SARS-CoV-2 using published estimates of weekly excess deaths and the country-specific, time-updated infection fatality rates (IFRs; S2 Table). We used crude excess mortalities and IFRs, which account for differences in the age structure of each population, but assume that infectious individuals are equally distributed across age groups within countries. Specifically, we used the central estimates from The Economist’s excess death model [47] and divided them by the IFR estimates from the COVID-19 Forecasting Team [48]. We sampled with replacement from the weekly estimated excess deaths and from a Normal distribution for the IFRs using the reported mean and 95%-confidence intervals. We then averaged each incidence sample across time periods and used these averages to estimate the risk of SARS-CoV-2 transmission for each country. The estimated incidences using our IFR-based approach are shown in Table 3. Since time-updated IFR estimates were only available until January 2021, our modeled transmission risks are for the early period of SARS-CoV-2, i.e. before widespread vaccination and the dominance of new SARS-CoV-2 variants such as delta and omicron. Note that by using weekly incidence as the prevalence of SARS-CoV-2, we implicitly assume a duration of infectiousness of one week.

### Sensitivity analyses

In the main analysis, we estimated the transmission risk of *Mtb* using the prevalence in the young population (age group 15–24 years). Because this age group has been found to have the most pronounced prevalence-to-notification gap [49], we performed a sensitivity check using the prevalence in the general population. Second, we compared the transmission risk of SARS-CoV-2 using the estimated incidence from our IFR-based approach with the transmission risk using the reported incidence [50, 51]. This comparison was only possible in Switzerland and South Africa, as national incidence was rarely reported in Tanzania [45, 46]. Third, we assessed the impact of assuming an outdoor CO_2_ level of *C*^*o*^ *=* 600 ppm instead of *C*^*o*^ *=* 400 ppm in each countries. Although many studies use *C*^*o*^ *=* 400 ppm [20, 22, 27–30], the outdoor CO_2_ levels can be *C*^*o*^ *=* 500 ppm or higher in urban areas, especially in the early morning, mainly due to traffic-related emissions [20].

### Additional analysis

In a further analysis, we considered an outbreak with one infectious person per classroom (one infectious person scaled to the class size) and estimated the weekly risk of transmission for both *Mtb* and SARS-CoV-2. In this hypothetical outbreak scenario, we also used the daily average rebreathed air fractions instead of overall average rebreathed air fractions to account for variations in daily ventilation.

### Ethics statement

Data from schools in South Africa and Tanzania were previously published [21, 22]. The Canton of Bern Ethics Committee reviewed and approved data collection from schools in Switzerland (KEK; reference no. 2021–02377).

## Results

### Ventilation of classrooms

**Fig 1:**
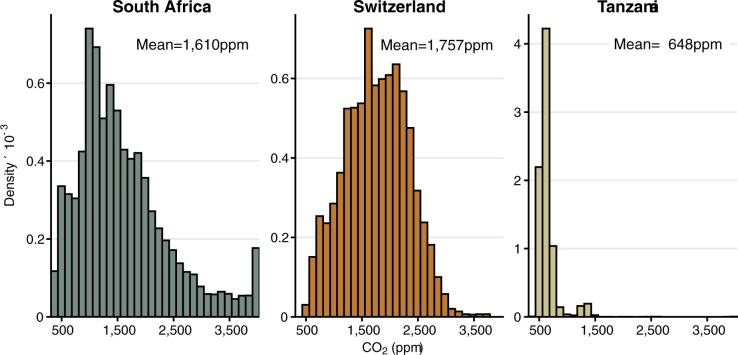
Carbon dioxide (CO_2_) levels in the classrooms of schools in South Africa, Switzerland, and Tanzania. Histogram of the measured CO_2_ levels (in parts per million [ppm]) in each country. All distributions are truncated at 400ppm (left) and 4,000ppm (right).

The ventilation of classrooms varied considerably between countries (Table 1). The average CO_2_ level was 1,610 ppm in South Africa, 1,757 ppm in Switzerland, and 648 ppm in Tanzania. These correspond to average rebreathed air fractions of 3.8% in South Africa, 4.3% in Switzerland, and 0.8% in Tanzania. There was considerable variation in CO_2_ levels over the study period (Fig 1). Based on the daily maximum CO_2_ levels, the ventilation rate was (mean±standard deviation [SD]) 2.47±1.81 L s^-1^ (1.48±1.08 air changes h^-1^) in South Africa, 2.29±0.88 L s^-1^ (0.71±0.27 air changes h^-1^) in Switzerland, and 12.36±7.46 L s^-1^ (13.74±8.29 air changes h^-1^) in Tanzania.

### Risk of *Mtb* transmission

**Fig 2:**
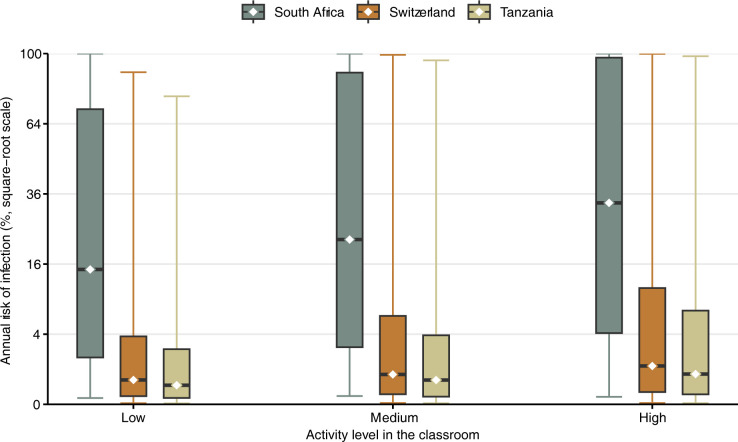
Modeled transmission risk of *Mycobacterium tuberculosis* (*Mtb*) in schools in South Africa, Switzerland, and Tanzania. Annual transmission risk (median as dots, interquartile range as boxes, and 95%-CrI as lines) of *Mtb*. Three scenarios are considered: low (70% breathing, 25% speaking, 5% loud speaking), medium (50% breathing, 40% speaking, 10% loud speaking), and high activity (30% breathing, 50% speaking, 20% loud speaking).

The annual transmission risk for *Mtb* is shown in Fig 2. Assuming medium classroom activiy, the median risk was 22.1% (interquartile range [IQR] 2.7–89.5%) in South Africa, 0.7% (IQR 0.1–6.4%) in Switzerland, and 0.5% (0.0–3.9%) in Tanzania. Assuming low or high activity, the median risk was 14.8% or 33.0% in South Africa, 0.5% or 1.2% in Switzerland, and 0.3% or 0.7% in Tanzania. Regardless of the assumption for the activity level, the 95%-credible intervals (CrI) typically ranged from 0% to 100% in all countries due to the wide range of the distribution of the infectious quanta generation rate (i.e. we accounted for large heterogeneity in patient infectiousness).

As a sensitivity analysis, we estimated the annual risk of *Mtb* transmission using the prevalence in the general population instead of the young population. In this case, the risk of infection would be higher in all countries, but the relative differences between countries remained similar (S1 Fig). We also examined the impact of assuming an outdoor CO_2_ level of 600 pm instead of 400 ppm. A higher outdoor CO_2_ level slightly reduced the estimated risk of transmission in all countries but the relative differences between countries remained similar even when the outdoor CO_2_ level would be 200 pm higher in one country than in the others (S2 Fig).

**Fig 3:**
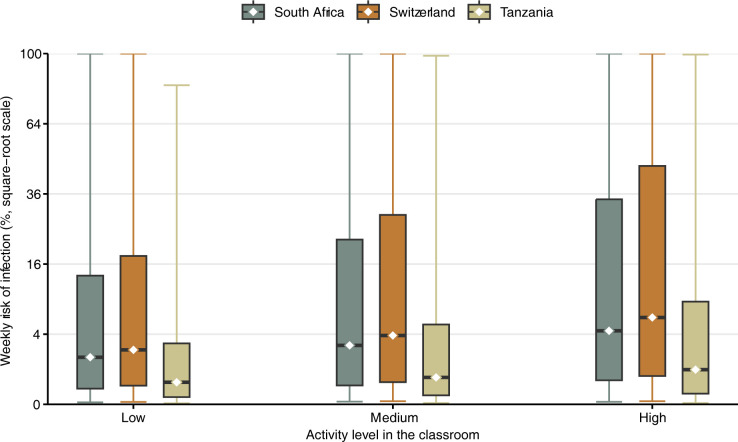
Modeled transmission risk during a hypothetical *Mtb* outbreak in schools in South Africa, Switzerland, and Tanzania. Weekly *Mtb* transmission risk (median as dots, interquartile range as boxes, and 95%-CrI as lines) if there was one infectious person per classroom (hypothetical outbreak scenario). Three scenarios are considered: low (70% breathing, 25% speaking, 5% loud speaking), medium (50% breathing, 40% speaking, 10% loud speaking), and high activity (30% breathing, 50% speaking, 20% loud speaking).

As an additional analysis, we assumed an outbreak with one infectious patient per classroom (hypothetical scenario shown in Fig 3). Assuming medium activity, the median risk of infection during a one-week outbreak would be 2.8% (IQR 0.3–23.5%) in South Africa, 3.8% (IQR 0.4–29.3%) in Switzerland, and 0.6% (IQR 0.1–5.3%) in Tanzania.

### Risk of SARS-CoV-2 transmission

**Fig 4:**
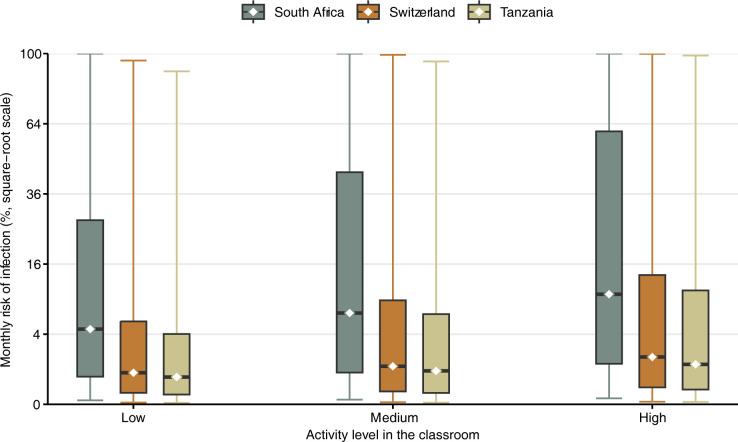
Modeled transmission risk of SARS-CoV-2 in schools in South Africa, Switzerland, and Tanzania. Monthly transmission risk (median as dots, interquartile range as boxes, and 95%-CrI as lines) of SARS-CoV-2. Three scenarios are considered: low (70% breathing, 25% speaking, 5% loud speaking), medium (50% breathing, 40% speaking, 10% loud speaking), and high activity (30% breathing, 50% speaking, 20% loud speaking).

The monthly transmission risk for SARS-CoV-2 is shown in Fig 4. Assuming medium classroom activiy, the median risk was 6.8% (interquartile range [IQR] 0.8–43.8%) in South Africa, 1.2% (IQR 0.1–5.6%) in Switzerland, and 0.9% (0.1–10.5%) in Tanzania. Assuming low or high activity, the median risk was 4.6% or 27.6% in South Africa, 0.8% or 1.8% in Switzerland, and 0.6% or 1.3% in Tanzania. Again, the 95%-credible intervals [CrI] ranged from 0% to 100% in all countries due to the wide range of the infectious quanta rate distribution.

As a sensitivity analysis, we estimated the monthly risk of SARS-CoV-2 transmission using the reported incidence in South Africa and Switzerland instead of the incidence estimated using our IFR-based approach. In this case, the risk of infection would be considerably lower in South Africa (S3 Fig), indicating significant underreporting of SARS-CoV-2 cases. Furthermore, and similar to *Mtb*, assuming a higher outdoor CO_2_ level in one country slightly reduced the risk of transmission but the relative differences between countries remained similar (S4 Fig).

**Fig 5:**
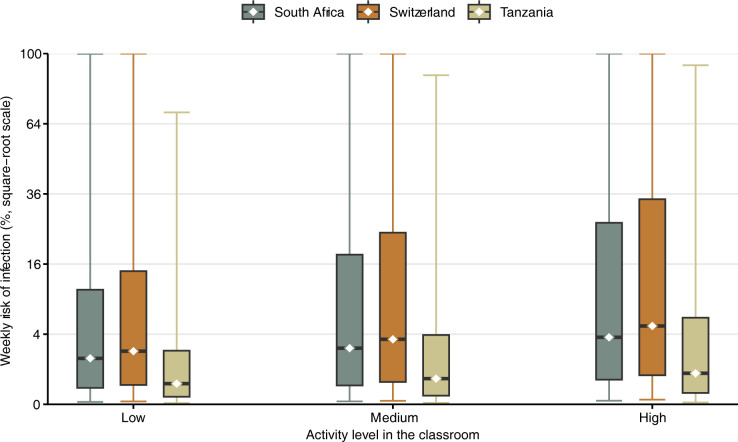
Modeled transmission risk during a hypothetical SARS-CoV-2 outbreak in schools in South Africa, Switzerland, and Tanzania. Weekly transmission risk (median as dots, interquartile range as boxes, and 95%-CrI as lines) of SARS-CoV-2 if there was one infectious person per classroom (hypothetical outbreak scenario). Three scenarios are considered: low (70% breathing, 25% speaking, 5% loud speaking), medium (50% breathing, 40% speaking, 10% loud speaking), and high activity (30% breathing, 50% speaking, 20% loud speaking).

Similar to *Mtb*, the risk of SARS-CoV-2 infection during a one-week outbreak with one infectious person per classroom would be more comparable between countries (hypothetical scenario shown in Fig 5). Assuming medium activity, the median risk of infection would be 2.6% (IQR 0.3–18.2%) in South Africa, 3.4% (IQR 0.4–24.0%) in Switzerland, and 0.8% (IQR 0.1–6.1%) in Tanzania.

## Discussion

We modeled the airborne transmission risk of *Mtb* and SARS-CoV-2 in secondary schools in South Africa, Switzerland, and Tanzania. The annual risk of infection for *Mtb* and SARS-CoV-2 during school was higher in South Africa than in Switzerland and Tanzania. Assuming a one-week outbreak with one infectious person per classroom, the risk of transmission in South Africa and Switzerland would be comparable, but still higher than in Tanzania because of better classroom ventilation in Tanzania.

A few caveats about our results. First, we model the transmission risk during school hours. The overall transmission risk for students may be higher because they may also be exposed to SARS-CoV-2 and *Mtb* outside of school. Second, the Wells-Riley equation only models the risk of airborne transmission, but other routes of transmission are possible. For example, some studies have suggested transmission of SARS-CoV-2 via droplets and fomites [52, 53], although their contribution to overall transmission is thought to be small [54–56]. Third, we modeled the infectious quanta generation rate *q* with a Lognormal distribution based on previous literature [33]. This distribution also covers a wide range of patient- and outbreak-specific estimates reported in the literature [33–38]. The variation in *q* is the result of variation in patient-specific infectiousness. As a result, the annual risk of transmission is typically low to moderate (see our IQR estimates), but can be extremely high (see our 95%-CrI estimates) if there is prolonged exposure to a highly infectious individual.

Several studies have modeled transmission risks of respiratory infections in indoor settings within countries [15, 21–23, 30, 57]. However, to our knowledge, comparisons of transmission risks in schools between countries are lacking. Our comparison of the risk of transmission between schools in different countries sheds light on the impact that different incidences of infectious diseases and natural ventilation have on the risk of infection with *Mtb* and SARS-CoV-2. The results are consistent with our previously published modeled estimates for Tanzania and South Africa [21, 22]. Empirical estimates for the risk of infection are scarce. Based on population-level observational data, the cumulative risk SARS-CoV-2 infection among students in Germany was shown to be between 1% and 10%, depending on the phase of the pandemic [12]. A systematic review concluded that the risk of infection following a SARS-CoV-2 outbreak in a high-incidence setting was 1.7–28% for school teachers, which was usually higher than for students [58]. Furthermore, seroprevalence was 2.1–6.2% in Swiss schoolchildren (aged 6–16 years) during June-September 2020 [59], and 0–7% in rural and 11–29% in urban South African adolescents (aged 13–18 years) during July-September 2020, respectively [60]. Seroprevalence studies for Tanzania were not available [61].

*Mtb* transmission is more difficult to estimate because of the lack of an appropriate in vitro test assay to measure infection [62]. Interferon-γ release blood tests (IGRAs) and tuberculin skin tests (TST) are commonly used to diagnose *Mtb* infection, but school-based surveys are rare. A recent study reported a TB infection incidence rate of 17.1 per 100 person-years among adolescents and young adults in South African communities of a cluster randomized trial [63]. The prevalence of TB infection among 12 to 18-year-olds is approximately 50% [64]. Using longitudinal IGRA data in a cross-sectional manner, the annual risk of infection has been shown to be as high as 14% [64]. In contrast to South Africa, which has one of the highest TB burdens in the world, the risk of infection in Tanzania is likely to be lower. Using IGRA data, the overall prevalence of TB in the 12 to 16-year-olds was estimated to be approximately 20% [65]. The annual risk of infection among 5 to 12-year-olds may be 5–10%, comparable to the risk in most high-burden countries [66], although survey data are lacking. A recent transmission modeling study calibrated to TB prevalence and notification data estimated the annual risk of *Mtb* infection to be up to 15% in South Africa and about 2.5% in Tanzania [67]. A similar modeling study using the Wells-Riley model with indoor CO_2_ levels and estimated TB prevalence found an estimated annual risk of infection of 11% in classrooms in KwaZulu-Natal, a high TB burden community in South Africa [57]. Overall, the empirical estimates for SARS-CoV-2 and *Mtb* are roughly in line with our modeled estimates, given the uncertainty surrounding these estimates. For *Mtb* in South Africa, however, estimates from the literature appear to be more consistent with our lower scenario estimates.

Environmental factors play an important role in the control of respiratory infections [68]. Previous studies have shown that inadequate ventilation facilitates the spread of *Mtb* in clinics and other public locations [21, 22, 30]. Based on our data, only Tanzania achieved air change rates above recommended levels [22]. Air change rates were below recommended levels in South Africa and Switzerland. Both countries ventilate their classrooms through window opening. Countries with limited resources may need to rely on natural ventilation and extend the times when doors and windows are open. In contrast, resource-rich countries can invest in improved building designs, mechanical ventilation systems, or hybrid solutions [69].

Our study has limitations. First, for SARS-CoV-2, we approximated the prevalence of infectious students in the room with disease incidence. While this approximation may bias the magnitude of the country-specific estimates, the biases will be in the same direction and thus have little impact on the comparison of transmission risks between countries. Second, we estimated incidence with a consistent approach using excess mortality and country-specific, time-updated infection-fatality ratios (IFR-based approach). On the one hand, this approach reduces substantial biases due to differences in reporting and testing practices between countries, which can also be seen in the lower transmission risk of SARS-CoV-2 when using the reported instead of the estimated incidence (S1 Fig). On the other hand, the estimated incidence was modeled and our IFR-based approach may still underestimate the true incidence because positive effects such as reduced road traffic may partly offset the effect of COVID-19 on excess mortality [70]. Third, due to data limitations, we could only model the transmission risk of SARS-CoV-2 for the early period through January 2021. For example, related work suggests that the viral load (and thus the quanta generation rate) are higher for the SARS-CoV-2 delta variant [71, 72].

The CO_2_ measurements were collected during different time periods (South Africa: 2013, Switzerland: 2023, Tanzania: 2015). However, all data were collected during non-pandemic times and conditions and the schools in South Africa and Tanzania were structurally still the same (no relevant building work, renovations, or improvements). Notably, no evidence for exogenous CO_2_ sources was found in the classrooms. Although systematic national survey data are lacking, the local investigators [21, 22, 26] considered the data collection to be representative of the overall situation in each country. Moreover, the CO_2_ levels in the Swiss school are very similar to those found in a recent survey of 100 classrooms in Switzerland [73], as well as in other European countries [74–78]. Fifth, we assumed a fixed number of students per classroom. The sensitivity of this assumption is difficult to assess because changes in room occupancy are likely to be accompanied by changes in CO_2_ levels, but to different degrees depending on the ventilation setting. Finally, our modeling approach assumed a well-mixed airspace and constant outdoor air supply. We acknowledge that environmental characteristics introduce spatiotemporal variation in the concentration of infectious particles and, correspondingly, in the risk of infection [2, 79, 80].

In conclusion, we modeled and compared *Mtb* and SARS-CoV-2 transmission risks in schools in South Africa, Switzerland, and Tanzania. Country-specific risks of infection primarily reflect differences in the number of infectious students and natural ventilation. Global comparisons identify high-risk settings and can inform prevention and mitigation strategies such as improved ventilation and other infection control measures.

## Supporting information

S1 Table Reported estimates in the literature for the infectious quanta generation rate (*q*) of *Mycobacterium tuberculosis* (*Mtb)*. (DOCX)

S2 Table Excess death estimates and time-updated, country-specific infection-fatality ratios (IFRs) used to estimate the incidence of SARS-CoV-2. (DOCX)

S1 Fig Sensitivity analysis showing the risk of *Mtb* transmission using the prevalence of *Mtb* in the general population. (DOCX)

S2 Fig Sensitivity analysis showing the risk of *Mtb* transmission risk assuming an outdoor CO_2_ level of 600 ppm in each country separately. (DOCX)

S3 Fig Sensitivity analysis showing the transmission risk of SARS-CoV-2 using the reported incidence of SARS-CoV-2. (DOCX)

S4 Fig Sensitivity analysis showing the transmission risk of SARS-CoV-2 assuming an outdoor CO_2_ level of 600 parts per million [ppm] in each country separately. (DOCX)
