# Supplementary material for: Airborne transmission risks of tuberculosis and COVID-19 in schools in South Africa, Switzerland, and Tanzania: Modeling of environmental data
Source: PLOS Glob Public Health. 2024 Jan 18;4(1):e0002800. doi: 10.1371/journal.pgph.0002800 (PMC10796007; doi:10.1371/journal.pgph.0002800)
Supplement: S1 Table — (DOCX) [file pgph.0002800.s001.docx]

**S1 Table. Reported estimates in the literature for the infectious quanta generation rate (*q*) of *Mycobacterium tuberculosis* (*Mtb)*.**

| **Author** | **Reported Estimation (quanta h^-1^)** | **Study type and study setting** |
| --- | --- | --- |
| Andrews *et al.*, 2014, Journal of Infectious Diseases, doi: 10.1093/infdis/jiu138 | Average of 0.89 across patients, assuming recurrent contacts and twelve months infectiousness. Lower estimate of 0.27 assuming no recurrent contacts and upper estimate of 5.69 assuming four months infectiousness (see their Supplementary Table S1) | Modeling study in the community of a South African township |
| Escombe *et al.*, 2008, PLOS Medicine, doi: 10.1371/journal.pmed.0050188 | Average of 8.2 across all patients. The range among patients identified as infectious was 1.8 to 226 (see their Table 2). Variability in infectiousness related to multidrug-resistant TB, treatment delay, and smear-positive/smear-negative test result. | Experimental study with guinea pigs in a hospital ward |
| Nardell *et al.*, 1991, American Review of Respiratory Disease, doi: 10.1164/ajrccm/144.2.302 | Estimate for a single outbreak of 12.7. | Modeling study in an office environment and one office worker with cavitary TB |
| Riley *et al*., 1961, American Review of Respiratory Disease, doi: 10.1164/arrd.1962.85.4.511 | Average of 1.25 but up to 60 for an extremely infectious patient, based on the computations of Nardell et al., 1991. | Experimental study with guinea pigs in a hospital ward |
