# Supplementary material for: Airborne transmission risks of tuberculosis and COVID-19 in schools in South Africa, Switzerland, and Tanzania: Modeling of environmental data
Source: PLOS Glob Public Health. 2024 Jan 18;4(1):e0002800. doi: 10.1371/journal.pgph.0002800 (PMC10796007; doi:10.1371/journal.pgph.0002800)
Supplement: S2 Table — (DOCX) [file pgph.0002800.s002.docx]

**S2 Table. Excess death estimates and time-updated, country-specific infection-fatality ratios (IFRs) used to estimate the incidence of SARS-CoV-2.** Excess deaths (deaths per 100,00 people) as officially reported or estimated by the The Economist’s excess death model (The Economist and Solstad, 8 Sep 2023, https://www.economist.com/graphic-detail/coronavirus-excess-deaths-estimates). Time-updated, country-specific IFRs as reported by the COVID-19 Forecasting Team (COVID-19 Forecasting Team, 2022, The Lancet, doi: 10.1016/S0140-6736(21)02867-1). Incidence (cases per 100,000 people) was computed as Excess deaths $\times$ IFR^-1^, based on 4,000 samples with replacement from the excess deaths data and 4,000 samples from the IFR distributions, respectively (see Materials and Methods for details). If the number of excess deaths was negative, incidence was set to 0.

| **Period** | **Excess deaths**  Median (95%-CrI) | **IFR**  Median (95%-CrI) | **Incidence**  Median (95%-CrI) |
| --- | --- | --- | --- |
| South Africa  Apr, 20  Jul, 20  Oct, 20  Jan, 21 | –1.57 (–3.01–0.63)  3.26 (–1.48–9.81)  1.00 (–0.66–2.80)  14.36 (5.47–25.27) | 0.511 (0.199–1.275)  0.400 (0.173–0.987)  0.348 (0.145–0.774)  0.331 (0.137–0.724) | 0 (0­­­­–246)  849 (0–9,096)  289 (0–2,122)  4,397 (1,083–25,578) |
| Switzerland  Apr, 20  Jul, 20  Oct, 20  Jan, 21 | 0.24 (–0.92–6.04)  0.05 (–0.97–1.10)  2.91 (0.04–10.13)  6.24 (2.07–10.24) | 1.687 (1.202–2.782)  1.332 (0.943–2.148)  1.164 (0.835–1.743)  1.113 (0.766–1.621) | 14 (0–472)  1 (0–102)  258 (0–1,208)  548 (145–1,224) |
| Tanzania  Apr, 20  Jul, 20  Oct, 20  Jan, 21 | 0.09 (–0.07–0.61)  0.86 (0.52–1.19)  1.19 (0.67–1.69)  1.87 (1.37–2.41) | 0.169 (0.073–0.339)  0.133 (0.060–0.256)  0.116 (0.057–0.218)  0.111 (0.052–0.193) | 64 (0–816)  644 (289–2,307)  1,048 (428–3,298)  1,738 (884–4,551) |
