# Supplementary material for: Airborne transmission risks of tuberculosis and COVID-19 in schools in South Africa, Switzerland, and Tanzania: Modeling of environmental data
Source: PLOS Glob Public Health. 2024 Jan 18;4(1):e0002800. doi: 10.1371/journal.pgph.0002800 (PMC10796007; doi:10.1371/journal.pgph.0002800)
Supplement: S1 Fig — (DOCX) [file pgph.0002800.s003.docx]

**S1 Fig: Sensitivity analysis showing the risk of *Mtb* transmission using the prevalence of *Mtb* in the general population.** Annual transmission risk (median as dots, interquartile range as boxes, and 95%-CrI as lines) of *Mtb* when using the prevalence of *Mtb* in the general population (left) instead of the prevalence in the age group of the 15 to 24-year-olds (right)*.* Only the medium activity scenario (50% breathing, 40% speaking, 10% loud speaking) is compared.

**
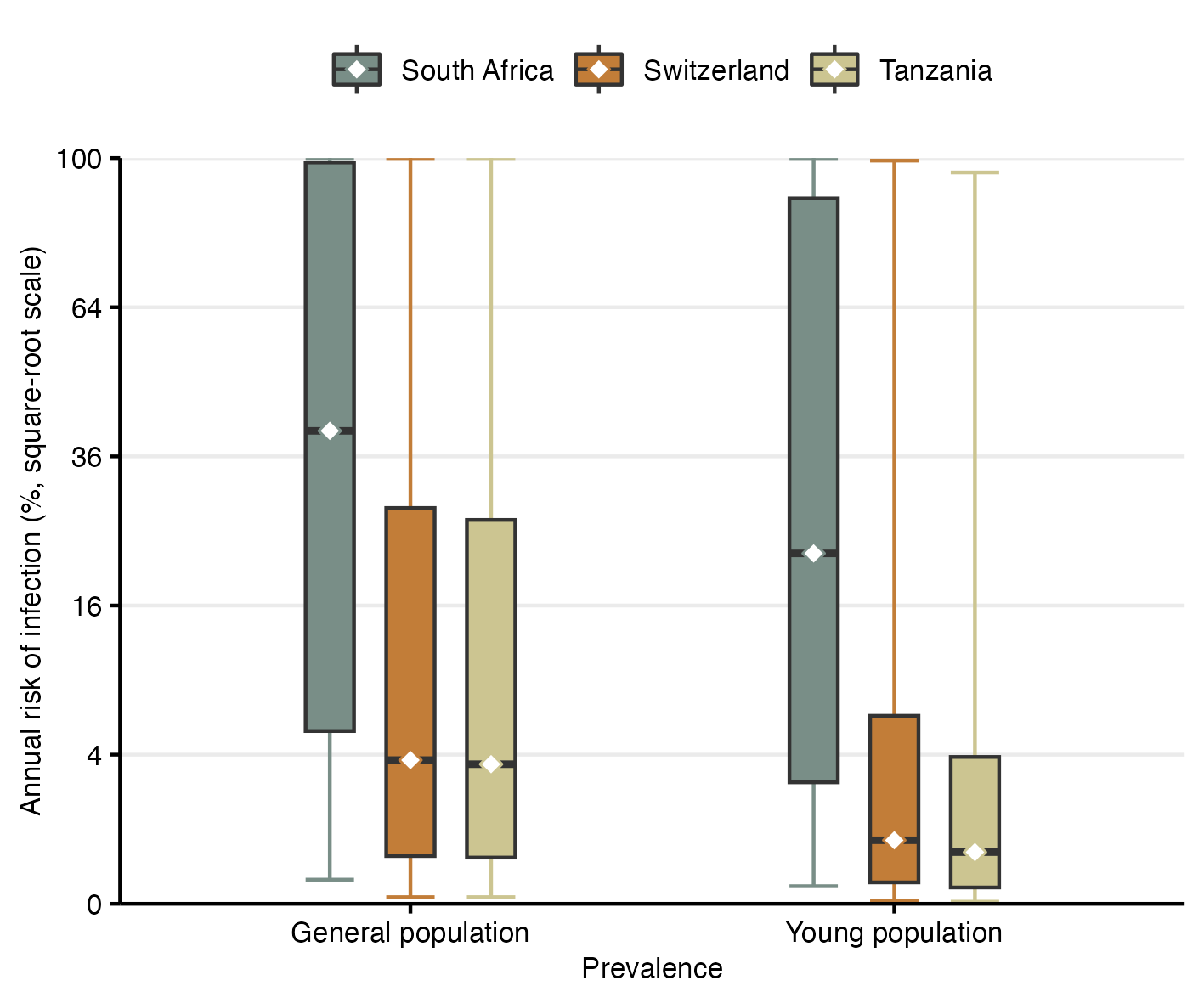
**
