# Supplementary material for: Airborne transmission risks of tuberculosis and COVID-19 in schools in South Africa, Switzerland, and Tanzania: Modeling of environmental data
Source: PLOS Glob Public Health. 2024 Jan 18;4(1):e0002800. doi: 10.1371/journal.pgph.0002800 (PMC10796007; doi:10.1371/journal.pgph.0002800)

**S2 Fig: Sensitivity analysis showing the risk of *Mtb* transmission risk assuming an outdoor CO_2_ level of 600 ppm in each country separately.** Annual transmission risk (median as dots, interquartile range as boxes, and 95%-CrI as lines) of *Mtb* comparing the annual transmission risk when assuming an outdoor CO­_2_ level *C^o^* = 600ppm in one country (dark triangle) vs *C^o^* = 400ppm in the other countries (white squares)*.* Only the medium activity scenario (50% breathing, 40% speaking, 10% loud speaking) is compared.


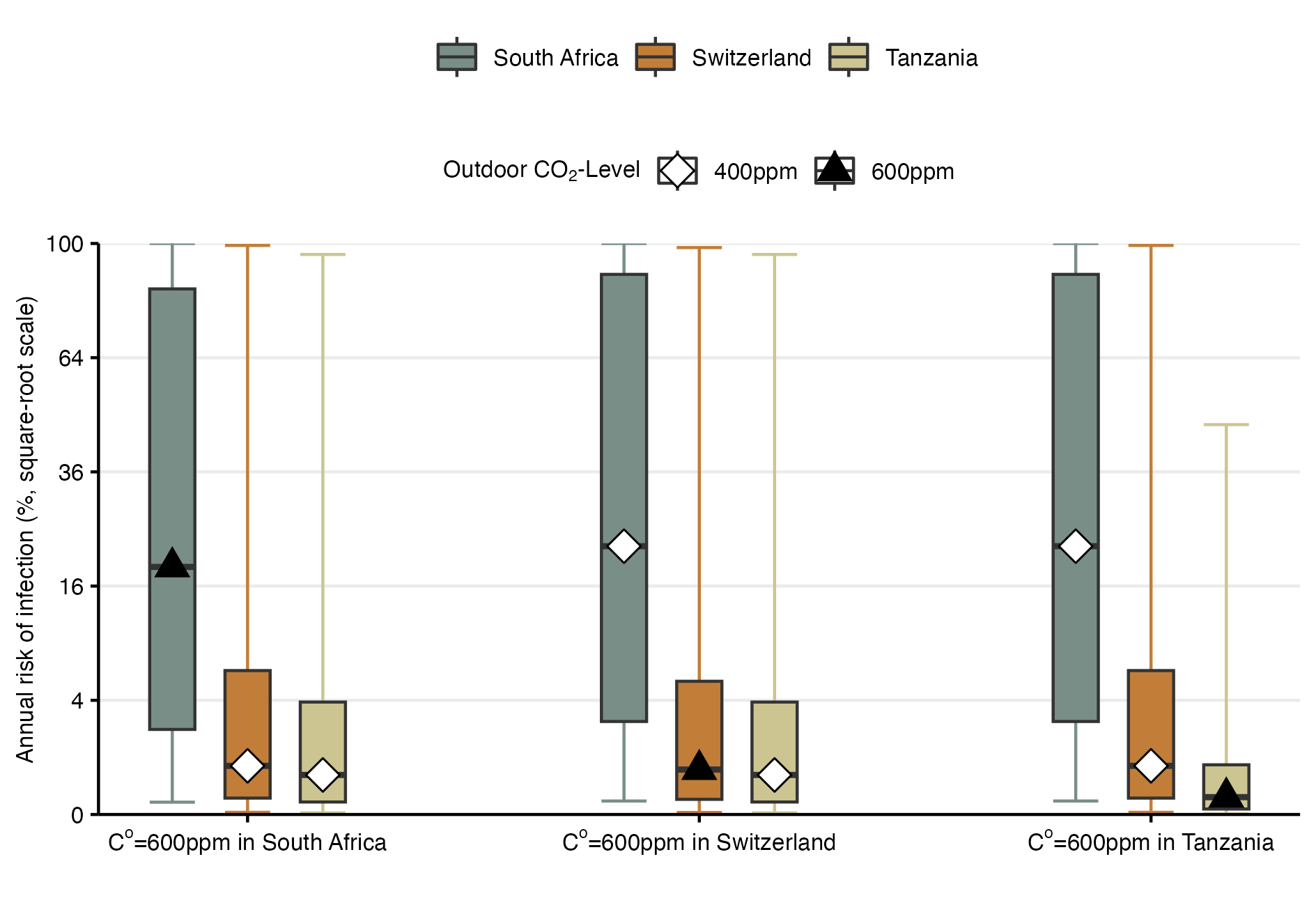

Supplement: S2 Fig — (DOCX) [file pgph.0002800.s004.docx]
