# Supplementary material for: Airborne transmission risks of tuberculosis and COVID-19 in schools in South Africa, Switzerland, and Tanzania: Modeling of environmental data
Source: PLOS Glob Public Health. 2024 Jan 18;4(1):e0002800. doi: 10.1371/journal.pgph.0002800 (PMC10796007; doi:10.1371/journal.pgph.0002800)

**S3 Fig: Sensitivity analysis showing the transmission risk of SARS-CoV-2 using the reported incidence of SARS-CoV-2.** Annual transmission risk (median as dots, interquartile range as boxes, and 95%-CrI as lines) of SARS-CoV-2 when using the reported incidence in the general population (left) or young population (middle) instead of the estimated incidence using our IFR-based approach (right)*.* Only the medium activity scenario (50% breathing, 40% speaking, 10% loud speaking) is compared. Incidence in Tanzania was barely reported (see Buguzi, 2021, BMJ, doi: 10.1136/bmj.n1052 and Murewanhema and Dzinamarira, 2022, International Journal of Environmental Ressearch and Public Health, doi: 10.3390/ijerph19084448) and thus not used. Reported incidence in the young population was only available for Switzerland.


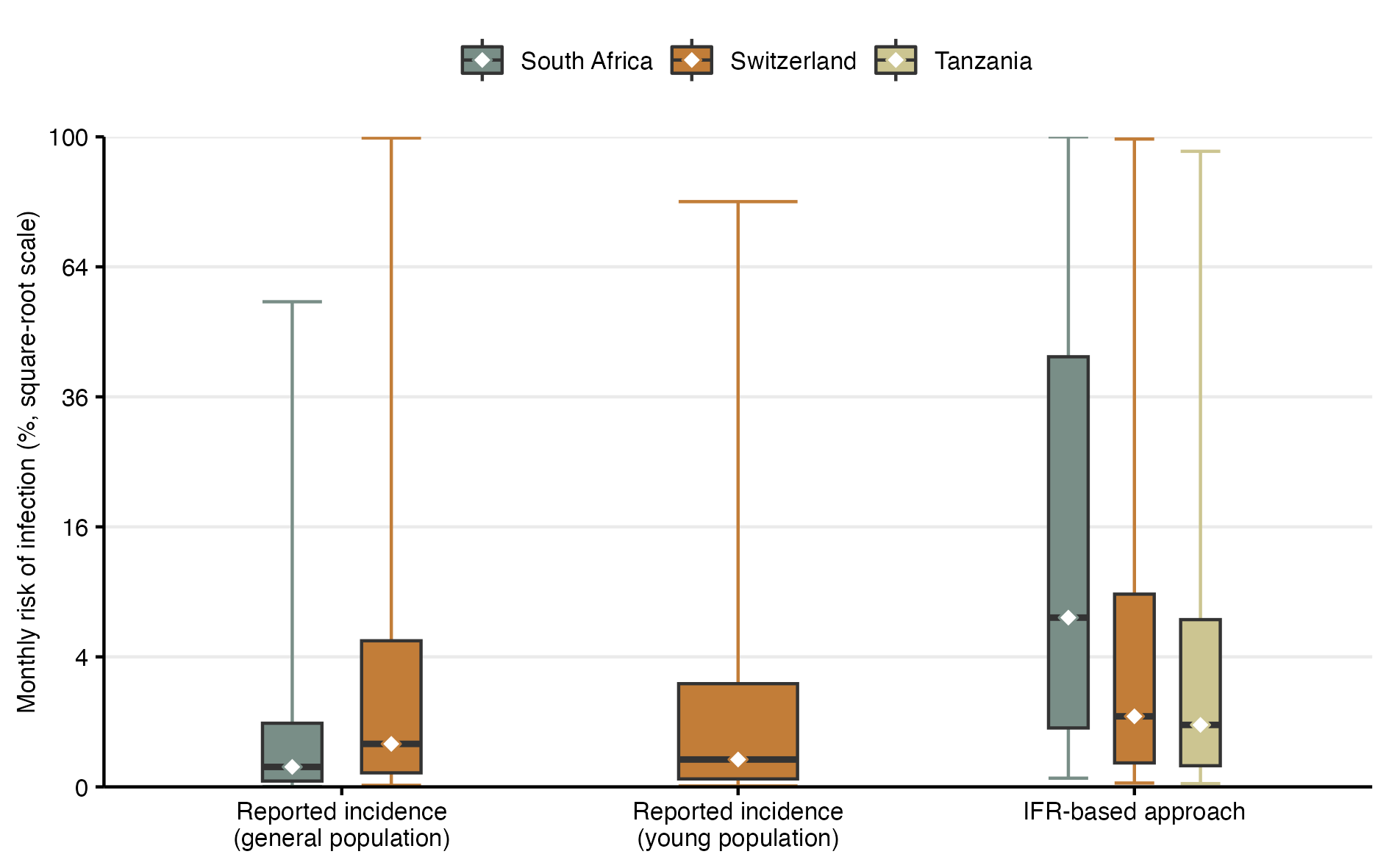

Supplement: S3 Fig — (DOCX) [file pgph.0002800.s005.docx]
